# Recent Progress on Ligand-Protected Metal Nanoclusters in Photocatalysis

**DOI:** 10.3390/nano13121874

**Published:** 2023-06-16

**Authors:** Meegle S. Mathew, Greeshma Krishnan, Amita Aanne Mathews, Kevin Sunil, Leo Mathew, Rodolphe Antoine, Sabu Thomas

**Affiliations:** 1 School of Energy Materials, Mahatma Gandhi University, Kottayam 686560, India; meeglesmathew@gmail.com (M.S.M.); grkgreeshmakrishnan@gmail.com (G.K.); amitamathews4@gmail.com (A.A.M.); kevin.sunil1@gmail.com (K.S.); leomathew97@gmail.com (L.M.); 2 Research and Post Graduate Department of Chemistry, Mar Athanasius College, Kothamangalam 686666, India; 3 Institut Lumière Matière UMR 5306, Univ Lyon, Université Claude Bernard Lyon 1, CNRS, F-69100 Villeurbanne, France

**Keywords:** photocatalysis, metal nanoclusters, CO_2_ reduction, hydrogen evolution reaction, photodegradation

## Abstract

The reckless use of non-replenishable fuels by the growing population for energy and the resultant incessant emissions of hazardous gases and waste products into the atmosphere have insisted that scientists fabricate materials capable of managing these global threats at once. In recent studies, photocatalysis has been employed to focus on utilizing renewable solar energy to initiate chemical processes with the aid of semiconductors and highly selective catalysts. A wide range of nanoparticles has showcased promising photocatalytic properties. Metal nanoclusters (MNCs) with sizes below 2 nm, stabilized by ligands, show discrete energy levels and exhibit unique optoelectronic properties, which are vital to photocatalysis. In this review, we intend to compile information on the synthesis, true nature, and stability of the MNCs decorated with ligands and the varying photocatalytic efficiency of metal NCs concerning changes in the aforementioned domains. The review discusses the photocatalytic activity of atomically precise ligand-protected MNCs and their hybrids in the domain of energy conversion processes such as the photodegradation of dyes, the oxygen evolution reaction (ORR), the hydrogen evolution reaction (HER), and the CO_2_ reduction reaction (CO_2_RR).

## 1. Introduction

Over the past several decades, the rising demands of the ever-growing population, excessive consumption of non-renewable resources, resulting greenhouse gas emissions, and improper waste disposal have greatly concerned humanity. The relentless efforts of the scientific community towards this global crisis have paved the way for more sustainable solutions such as photocatalysis. Photocatalysis appears to be a perfect fit as a greener alternative to resolving energy- and pollution-related problems simultaneously, and it aids the completion of chemical processes with the help of inexhaustible solar power, mitigates hazardous products such as CO_2_ by photoreduction, and produces cleaner fuels. In 1972, Fujishima and Honda were the first to apply this strategy for the hydrogen evolution reaction through photocatalysis [1]. Later, photocatalysis with semiconductors with sufficient bandgaps (i.e., TiO_2_, CdS, ZnO, Fe_2_O_3_, and ZnS) did turn into a promising field of study for researchers [2,3,4,5,6]. Nanotechnology has created revolutionary changes in this dimension with the help of umpteen nanoparticles (NPs), which possess a high surface area and plasmonic resonance. Noble metal nanoparticles, such as AuNPs, AgNPs, PtNPs, IrNPs, OsNPs, RhNPs, and RuNPs, have been employed mainly for photocatalysis for decades, but its efficiency has not reached the target level [7]. Apart from this, the mechanism-related information in photocatalysis driven by noble metal NPs with massive surface atoms does seem vague. Meanwhile, a new class of zero-dimensional fluorophores, MNCs, have proved to be dominant to their conventional nanoparticle analogs in various applications.

MNCs comprise a core with ten to hundreds of atoms protected by surface ligands such as thiols, proteins, peptides, enzymes, polymers, and DNA [8,9,10,11]. The emergence of these well-defined aggregates with commendable surface-to-volume ratios, fully reduced atomic cores, and dispersity on catalysts has made them the best choice for a wide range of applications [12]. Compared to bulk, because MNCs are sub-nanometer-sized units (<2 nm), they exhibit quantum confinement effects as they approach the Fermi wavelength of conduction electrons, thereby splitting the continuous density of states into discrete energy levels. As a result, MNCs possess molecule-like behavior and act as a missing link between atoms/molecules and metal nanoparticles [13]. The atomic-level precision of MNCs assists with confining their size and makes analyzing their involved mechanisms quite understandable. Due to these factors, they have also been assigned a few other titles, such as quantum clusters (QCs) and monolayer-protected clusters [14,15]. Initially, gold nanoclusters (AuNCs) were the focus of this study due to the simple synthetic routes, commendable stability and novel optical properties presented, even in the absence of plasmonic resonance, which are all attributed to their shift in valency from Au (III) to Au (I)/Au(0) in AuNCs [16].

**Figure 1:**
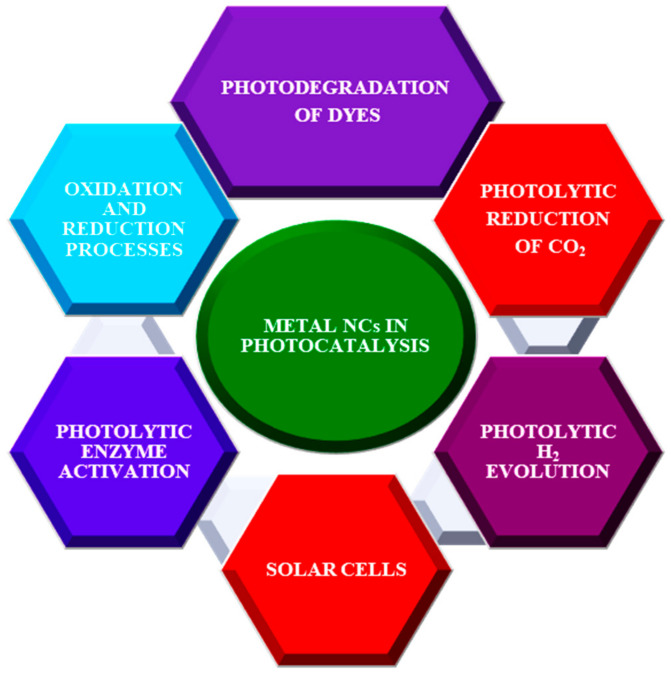
Applications of MNCs in photocatalysis.

The physicochemical properties of NCs are highly influenced by their size, metal core composition, assembly architecture, and surface components [17,18,19]. In addition, MNCs are known for their attractive optical characteristics, including a tunable luminescence, HOMO–LUMO transitions, a substantial Stokes shift, two-photon absorption, photostability, magnetism, chirality, and biocompatibility [20,21]. Their photostability, biocompatibility, and low cytotoxicity, in turn, have caused them to be chosen for biomedical applications [22]. Monolayer-protected clusters are less aggressive and relatively more stable than gas-phase clusters containing unsatisfied valence electrons in their free state. Currently, further studies on these factors and their impacts on optical properties have set forth new developments in catalysis, biosensing, bioimaging, gene therapy, and drug delivery [13,23,24,25,26]. Tailoring a cluster’s distinctive optical and electronic properties by enhancing parameters such as its formal charges, geometry, metal composition, and ligand plays a pivotal role in photocatalysis [27]. Despite this, MNCs with fully reduced metal atom cores have relatively low band gaps, thus inhibiting their photo-corrosion appreciably. Therefore, MNCs can be utilized as photosensitizers and cocatalysts in energy-intensive processes such as the photodegradation of dyes, ORR, HER, and CO_2_RR [28,29,30,31] (Figure 1).

A comprehensive review of metal nanomaterials for heterogeneous catalysis was written by Liu and Corma [12]. Jianping Xie and colleagues highlighted in a minireview the major important characteristics of MNCs, which are vital to photo- and electro-catalysis [27]. This review aims to update the literature on MNCs—their synthetic routes, physicochemical properties, stability, and recent advances, with a focus on photocatalysis, particularly from 2016 onwards, since a comprehensive review on the catalytic applications of ligand-protected, atomically precise MNCs was published in the Coordination Chemistry Journal in 2016, to which the readers refer to [32].

### 1.1. Chemical Composition and Structural Properties

Their central metal atom, ligands, charge states, and composition play a significant role in deciding the physicochemical properties of MNCs. In general, MNCs are expressed by the molecular formula [M_n_(SR)_m_]^q^ (n—represents the number of metal atoms, m—represents the thiolate ligands (staple motif), and q denotes the net charge of the cluster) [33]. Engineering the metal, ligand, and charge state on an atomic level can alter the performances and physicochemical properties of MNCs. In addition, their size (~2 nm) and structure can be adjusted with atomic precision for new application possibilities in various disciplines [34]. Using an analogy with the terminology employed in the protein field, MNCs are composed of primary, secondary, and tertiary structures. The metallic core is the primary structure and the repetitive local structural motifs serve as a bridge between the core and the ligands. These motifs are organic ligands, and surprisingly, their length, size, or structure can be easily manipulated, resulting in a wide range of MNCs. The protective shell’s exterior structure is made up of spatial ligands. It has been highlighted that insight into the crystal structure of the material is very crucial, as it reveals information on the atomicity of the core, the nature of the Au–S linkage, the chirality of the NCs, the arrangements of the ligands around the metal core, and so on [35]. In recent times, the crystal structures of some of the AuNCs, such as Au_102_SR_44_, [36] Au_25_SR_18_, [36] Au_38_SR_24_, [37] Au_36_SR_24_, [38] [Au_24_ (PPh_3_)_10_ (SR)_5_ Cl_2_]^+^, [39] and Au_28_SR_20_, [40] (where SR = thiolate) were resolved using various analytical techniques [41].

The molecular weight of MNCs is identified using mass-spectrometry-based methods, such as matrix-assisted laser-desorption ionization-time of flight mass spectrometry (MALDI-TOF-MS) and electrospray ionization mass spectrometry (ESI-MS) [42,43]. For MALDI analyses, traditional weak organic acids are used as matrices to recognize the molecular mass of the core of the MNCs; even though it is a sophisticated analytical instrument, it also has some limitations. The weak organic acid matrices employed in MALDI do not eliminate ligand fragmentation. The mass accuracy of MS equipment is also decreased by the presence of significant chemical noise and/or an unresolved isotopic pattern, which leads to the possibility of numerous molecular formulae being nearly isobaric and the potential for impurities or ligand fragmentation to produce significant chemical noise that makes it difficult to assign atomic information. For instance, isotope-resolved mass spectrometry has been pushed forward by Antoine’s group in order to unravel the molecular formula of ultrasmall NCs [44].

### 1.2. Synthesis of Metal Nanocluster

#### 1.2.1. General Synthetic Methods

A uniform, monodisperse catalyst with a known structure and composition should be desirable for the development of an effective catalyst. There are several synthetic techniques that can be used to produce luminescent metal nanoclusters with various sizes, structures, and surface characteristics. The conventional methods for producing these luminescent metal nanoclusters are various template methods, the photoreduction method, the sonochemical method, the microemulsion method, the radiolytic method, the electrochemical method, the microwave-assisted synthesis seed growth method, the monolayer-protected method, phase transfer synthesis, and the etching method, etc., In general, nanocluster synthesis can be categorized as a top-down approach, bottom-up approach, and inter-cluster conversion approach [45].

#### 1.2.2. Bottom-Up Method

The bottom-up method uses metal salt, ligands, and reducing agents as precursors. In this method, nanoclusters are formed via a wet chemical reduction of metal salts with a suitable reducing agent. In the first stage, a metal(I)–thiolate complex is formed by reacting metal salt with a thiolate ligand. Then, the metal(I)–thiolate complex is treated with a reducing agent, such as sodium borohydride (NaBH_4_) or ascorbic acid, to reduce M(I) to M(0) and produce M(0)@M(I)-based NCs [46]. This method of synthesis is also known as one-step synthesis. Nigeshi et al. synthesized and isolated a series of nanoclusters with different compositions, (Au_10_(SG)_10_, Au_15_(SG)_13_, Au_18_(SG)_14_, Au_22_(SG)_16_, Au_22_(SG)_17_, Au_25_(SG)_18_, Au_29_(SG)_20_, Au_33_(SG)_22_, and Au_39_(SG)_24_), using glutathione (SG) as stabilizing agent [47].

In some circumstances, the stabilizing ligand itself serves as a reducing agent, which eliminates the need for a second reducing agent. This technique of synthesis is known as the biomineralization method [48]. Other than wet chemical reduction, the photoreduction method is also employed to produce luminescent metal nanoclusters, which initiate the reduction reaction in light. Zhou et al. employed the photoreduction method for the synthesis of AuNCs stabilized by silane. The silane-stabilized nanocluster was further used for the photodegradation of an organic dye, methene blue [49].

#### 1.2.3. Top-Down Method

In the top-down approach, ultra-smaller-sized MNCs can be prepared from larger-sized metal nanoparticles/MNCs via chemical etching. Here, chemical etching is carried out between excess ligands and larger metal nanoclusters to obtain metal NCs with smaller sizes. This is usually performed in the solution phase, either in one solution or in an interface of two solutions. Based on the use of an etching agent, the etching method is classified into two categories, ligand etching and solvent etching. One major advantage of nanoclusters produced by the etching method is their controlled size focusing [50]. This method not only provides monodisperse metal nanoclusters, but also produces alloy nanoclusters [51,52].

#### 1.2.4. Inter-Cluster Conversion Method

In the inter-cluster conversion method, NCs are formed via seed-mediated synthesis, cluster conversion, metal exchange ligand exchange, and motif exchange. In this process, nanoclusters are used as the starting material and the structures of these nanoclusters are changed through an adjustment of their kinetic or thermodynamic parameters. The most common method for cluster conversion is the ligand exchange reaction (LER) [53]. The LER is a widely used technique for modifying nanoclusters after their creation. The adaptability of the gold and sulfur interphase makes this possible. The ability to change the sizes and phases of clusters and impart fluorescence onto nanoclusters for biological labelling purposes are some advantages of the LER. They can also increase the enantiomeric excess of already chiral clusters and give chirality to nonchiral clusters. In this way, the LER broadens the range of MNCs by forming distinctive and precise nanoclusters [54]. Wang and co-workers summarized the various LERs on thiolate-protected gold nanoclusters and their advantages [54,55,56].

Bootharaju et al. developed a procedure for the reversible transformation of NCs with different sizes. A reversible transformation of [Ag_35_(SG)_18_ to Ag_44_(4-FTP)_30_] or the shrinkage of [Ag_44_(4-FTP)_30_ to Ag_35_(SG)_18_] were carried out using the ligand SG and 4-fluorothiophenol (4-FTP) [57]. Similarly, an electrochemical method for the crystallization of NCs was put forward by Antonollo et al. [58]. Using this method, a large quantity of high-quality crystalline Au_25_(SR)_18_ NCs can be obtained. This method of crystallization can aid in determining the structure of new NCs, enabling a deeper comprehension of their molecular physiochemical characteristics.

The following section discusses certain widely used methods for producing metal nanoclusters.

#### 1.2.5. Monolayer-Protected Method

The monolayer-protected method is a simpler, direct, and universal method of producing uniform-sized metal nanoclusters. This method was first introduced by Burst et al. in 1994 for the synthesis of metal nanoparticles protected by monolayer mercaptan ligands [59]. The method uses a two-phase method to extract Au(III) chloride, control the molar ratio of thiol molecules to Au(III) chloride, and directly synthesize monolayer-protected gold nanoclusters. Followed by this invention, the Brust–Schiffin method has been widely used for the synthesis of various nanoclusters stabilized by thiol. This method has also been named the “direct synthesis method”. Tsukuda et al. used this method to synthesize and separate a series of SG-stabilized AuNCs, (Au_10_(SG)_10_, Au_15_(SG)_13_, Au_18_(SG)_14_, Au_22_(SG)_16_, Au_22_(SG)_17_, Au_25_(SG)_18_, Au_29_(SG)_20_, Au_33_(SG)_22_, and Au_39_(SG)_2_., by adopting the Busrt–Schiffin method [60]. One of the drawbacks of this method is its low product yield [61].

#### 1.2.6. Etching Method

The etching method is one of the major top-down methods for the synthesis of precise MNCs. By utilizing a suitable etching agent, larger metal particles can be etched into NCs of a precise size. The etching method is classified into ligand etching and solvent etching based on the selection of the etching agent [45]. Edinger et al., for the first time, reported the etching property of mercaptan. They found that mercaptan has the ability to remove the Au atoms from the surface of gold nanoparticles and further etch them to form AuNCs [62]. Followed by this invention, the etching strategy has been widely exploited for the synthesis of various NCs. Pradeep et al. synthesized used Au_25_ and Au_8_ from mercaptosuccinic-acid-protected larger-sized gold nanoclusters using SG as the etching agent [63]. Xie et al. developed a solvent etching method for the synthesis of MNCs. In this method, they altered the hydrophilic and hydrophobic properties of the clusters using electrostatic adsorption, then transferred the clusters from the aqueous phase to the organic phase, etching them under a mild reaction to obtain MNCs with a uniform size distribution [64].

#### 1.2.7. Template Method

The template method is considered to be one of the most popular bottom-up synthetic strategies for MNCs in recent times. In this method, ligands are used as reducing and stabilizing agents for the preparation of NCs. The templates used to produce NCs are peptides, proteins, polymers, dendrimers, DNA, and enzymes, etc. The protocols for the synthesis of different ligand-stabilized nanoclusters, their properties, and applications are discussed in the text book entitled “Luminescent Metal Nanoclusters-Synthesis Characterization and applications” [65]. Depending on the ligands used for the stabilization, the properties and structures of nanoclusters vary. The aromatic amino acid present in the macromolecule acts as reducing agent and cysteines and amino groups will stabilize the nanocluster. The following section will discuss the trends in ligands used for the synthesis of nanoclusters

#### 1.2.8. Trends in Ligands Used for Nanocluster Stabilization

**Figure 2:**
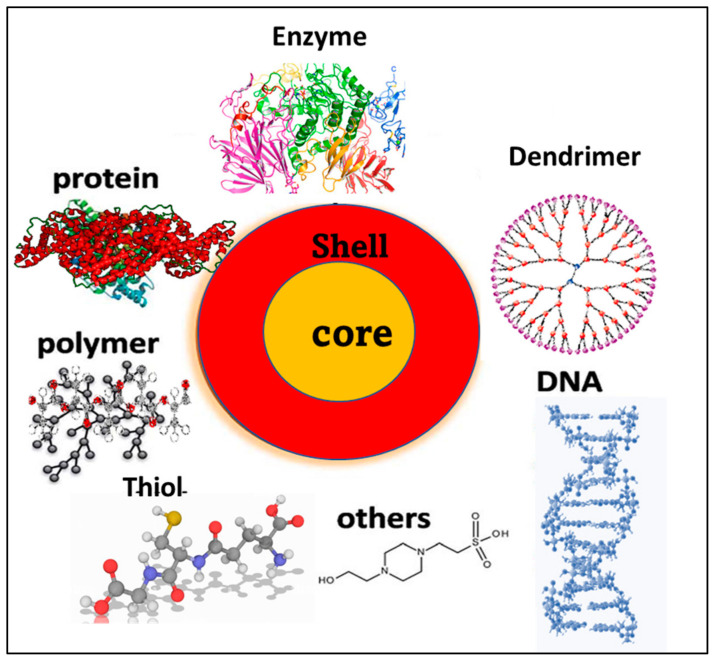
Shows a schematic representation of the ligands used for MNCs synthesis. (core: metal; shell: ligand).

Nanocluster chemistry starts from gas-phase clusters. The gas-phase cluster is the first reported nanocluster, where MNCs are formed via evaporation, and it is unprotected. These unprotected clusters are observed to be very reactive and form larger-sized particles [66]. Therefore, proper stabilization techniques should be used for MNC synthesis. The selection of ligands is an important step in the controlled synthesis of these MNCs. The first ligand chosen for the synthesis of MNCs was phosphine, due to its high affinity towards metal ions. Briant et al. developed a synthetic strategy for the preparation of icosahedral [Au_13_-(PMe_2_Ph)_10_C_l2_](PF_6_)_3_ [67]. Followed by a phosphine-stabilized cluster, various thiol-stabilized NCs have been reported due to the relatively high affinity of sulfur towards metal ions. Due to the insolubility of organic thiol-stabilized NCs in water, water-soluble thiols were introduced to synthesize NCs. Thiol-stabilized clusters were first introduced by Whetten et al. [68]. The ligand used for their stabilization was SG, and thiol molecules such as phenylethanethiol, hexanethiol, octanethiol, and dodecanethiol- mercaptosuccinic acid (MSA), etc., were used for the stabilization of nanoclusters by taking the advantages of thiol-gold chemistry [69]. Later, new possibilities were raised for creating quantum clusters using a macromolecule template. In this regard, macromolecules, such as various DNA, protein, polymers, and dendrimers, have been used for cluster stabilization and protection [65,70]. The template or ligand used for the stabilization must have a high binding affinity towards the metals (Au, Ag, and Pt, etc.) in order to prepare highly stable AuNCs with a high monodispersion [71]. Figure 2 depicts the core–shell nature of MNCs, which can be prepared by selecting the appropriate capping ligands [72]. The nanoclusters can be synthesized in different ways, such as via chemical reduction, photoreduction, hydrothermal, biomineralization, and etching, etc. [73].

Xie et al., for the first time, developed a biomineralization strategy for synthesizing highly luminescent gold nanoclusters, using BSA as protecting and reducing agent [74]. Red luminescent AuNCs comprise 25 gold atoms (Au_25_). The same strategy is used for the synthesis of various other protein-protected metal NCs [47,75]. Recently, Mathew et al. synthesized a highly stable fluorescent gluten-stabilized nanocluster. Gluten is a high-molecular-weight protein derived from wheat; it exhibits an enhanced stability towards reactive oxygen species [48].

The reaction duration, pH, temperature, type of ligand, template structure, reducing agent’s concentration, and Au^3+^/ligand ratio are crucial synthetic parameters for influencing the structure, size, surface characteristics, oxidation state, and, consequently, optical properties of MNCs [48].

### 1.3. Key Physicochemical Properties of MNCs in Photocatalysis

MNCs with specific structural designs and resultant exceptional physicochemical, electronic, and optical properties have given birth to a plethora of distinctive materials capable of driving photochemical reactions. In particular, tailoring their optical and electronic parameters could effectively alter their light-harvesting abilities and render a method for manipulating certain photocatalytic processes by inducing the generation of electron-hole pairs to attain the maximum quantum yield. For photocatalysis, the major criterion for choosing materials is a strong absorption of solar light [76]. It is worth noting that the sunlight that reaches the Earth consists of 3% UV light (280–400 nm), 45% visible light (400–800 nm), and 52% NIR (and IR) light (800–2500 nm). Therefore, to make full use of this solar energy, it is very important to improve the light-harvesting capability of semiconductors in visible and NIR regions. This can be realized by designing efficient Au-modified photocatalysts. Both metal nanoparticles and MNCs serve as photocatalysts for solar-energy-harvesting applications.

On this basis, the deposition of Au nanomaterials on the surface of semiconductors provides an effective way of enhancing their light-harvesting capabilities, especially for materials with a large optical gap, which therefore cannot absorb visible light. Due to the strong localized surface plasmon resonance of AuNPs, AuNP-modified photocatalysts can exhibit a remarkable light absorption enhancement in the visible light region. Similar to AuNPs, a few atom MNCs feature a suitable highest occupied molecular orbital (HOMO)–lowest unoccupied molecular orbital (LUMO) gap for visible and even near-IR light absorption and a long lifetime of excited states for efficient charge separation, making them emerging candidates for solar-energy-harvesting applications. The type of MNC, its size, and its ligands are fundamental determining factors for its responses to the wavelength of the absorbed light, adding versatility to photo absorption properties as compared to plasmonic particles.

**Figure 3:**
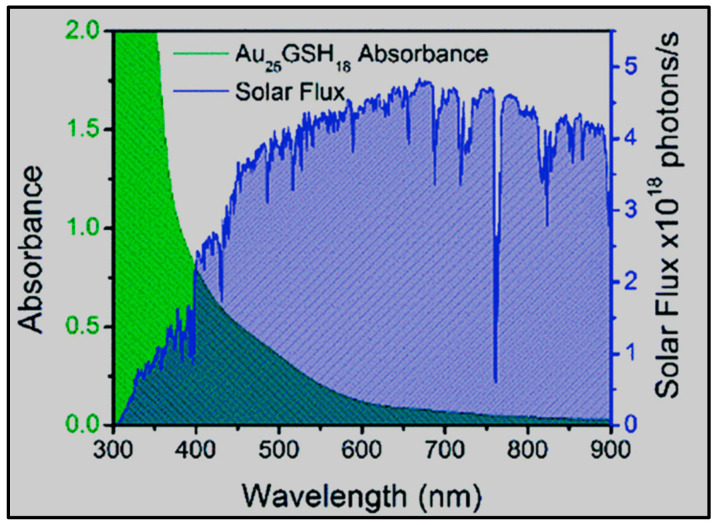
UV-Vis absorption spectrum of Au_25_GSH_18_ and solar flux for AM 1.5. (Xenon lamp was employed as the solar radiation source). Reprinted with permission from [76]. Copyright 2012 Royal Society of Chemistry.

Figure 3 shows the absorption spectrum and solar cells excited with AM 1.5 (100 mW cm^−2^) of Au_25_GSH_18_ (where GSH is reduced glutathione). The overlapping region between these two curves is a key component of photocatalysis. For AuNCs, efficient light energy conversion necessitates a slow rate of excited state relaxation. Indeed, Au_18_GSH_14_ exhibits the highest electron transfer rate and longest excited state lifetime of the NC series [76].

### 1.4. Optical Properties

#### 1.4.1. Optical Absorption

**Figure 4:**
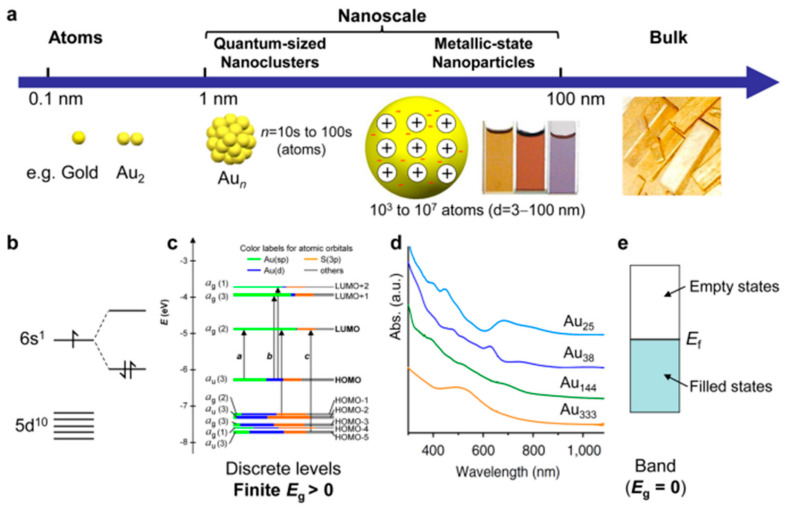
Elucidates the significant distinction between the atomic and bulk regimes concerning size and optical properties. (**a**) The nanoscale (1–100 nm) exhibits two distinct size regimes (quantum-sized: 1–3 nm (tens to hundreds of atoms), and regular metallic-state nanoparticles: 3–100 nm). (**b**) Atomic and diatomic electronic states. (**c**) Molecule-like electronic structure in quantum-sized nanoclusters (where, HOMO = highest occupied molecular orbital, LUMO = lowest unoccupied molecular orbital, *E*_g_ = HOMO-LUMO gap). (**d**) Evolution from discrete electronic excitation to collective electron excitation (plasmon) in optical absorption spectra with increasing size of nanoclusters. (**e**) Continuous band electronic structure of metallic-state nanoparticles and bulk metals (where, *E*_f_ = Fermi level/energy).Reprinted with permission from [80] Copyright 2021 Nature.

Metal nanoparticles, which have a size range from ~3 nm to 100 nm, obey the plasmonic regime, where a single surface plasmon resonance band dominates the optical spectrum, which has a far higher number of atoms than the number of surface molecules that stabilize it (see Figure 4). In such a regime (plasmonic regime), nanoparticles interact with the incident light and surface plasmon resonance occurs, causing conduction band electrons to collectively oscillate and form characteristic peaks (gold nanoparticles show surface plasmon resonance at 520 nm) [77,78]. On the contrary, NCs have a very small number of metal atoms (such as 2, 8, 18, 25, and 55, etc.), resulting in an optical spectrum with several bands, distinctive energy levels, and quantum behavior [79].

Figure 4 depicts the electrical transitions between the discrete energy levels in MNCs [81,82]. As the particle diameter decreases, SPR peaks disappear gradually, as shown in Figure 4. The Kohn–Sham orbital energy diagram of Au_25_SR_18_ nanoclusters indicates a HOMO composed of triply degenerated 5d10 atomic orbitals and doubly degenerated LUMO, which are 6sp atomic orbitals of gold [83] (Figure 4). Peak “a” formed due to the HOMO (sp) to LUMO (sp) and peak “b” is a mixed-type band (sp to sp and d to sp) transition. Peak “c” formed due to the (d to sp) inter-band transition (Figure 4). Moreover, the absorption of the nanoparticle depends on the metal nanoparticle size. There are several factors that depend on the photocatalytic activity of the catalyst, such as an increased visible light absorption, a smaller size, a higher surface area, and the optimum band gap, etc. It is reported that the photocatalytic activity of the nanoparticle, as well as the NCs, increases with a decreasing size of the particle, even though they are absorbed in the visible or UV region.

Recently, Rongchao et al. carried out a systematic analysis of the size effect on NCs and nanoparticles for photocatalysis. Generally, smaller NCs have larger specific surface areas and thus more catalytic active sites than larger NCs. The same observation has also been noticed in the case of larger metal nanoparticles [84,85].

#### 1.4.2. Photoluminescence

**Figure 5:**
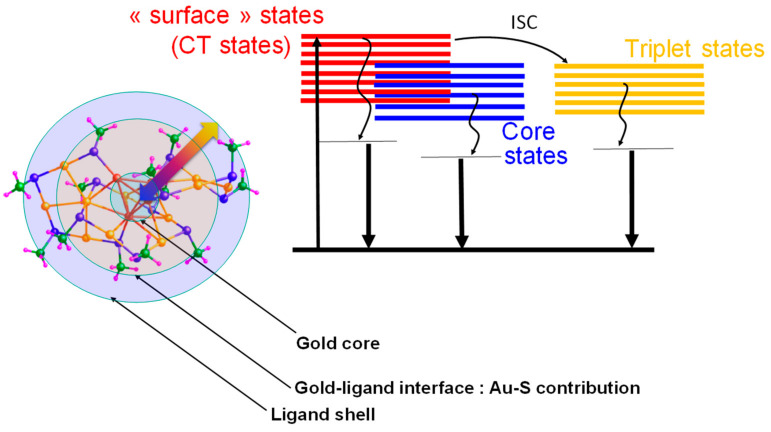
Schematic representation of the relaxation pathways of ligand-stabilized atomically precise AuNCs. Illustration of structural bonds and the metal–ligand interface by bringing in AuNCs as an example.

The photoluminescence property of MNCs opens up possibilities and applications in a variety of fields, including sensing and imaging, etc. [48,86]. The luminescence in MNCs depends on the geometric and electrical structures of their metal cores, as well as their ligand shells, and with an alteration to the metal core or ligands, one can tune their optical properties. Such NCs can be thought of as “multi-shell systems,” comprising a metallic core containing a metal–metal bond, a metal–ligand staple motif as an interface, and surface ligand molecules (see Figure 5). As observed in metal complexes, metal nanoclusters also exhibit similar charge transfer or electron transfer processes. These three shells can interact with each other via ligand-to-metal core or ligand-to-metal-metal charge transfers or by directly donating delocalized electrons from the ligands’ electron-rich groups [11]. Certain broad tendencies in de-excitation routes have been discovered following visible or near-UV absorption. The experimental and theoretical data from the literature were used to produce the energy diagram in Figure 5. As described in Figure 5, ligand-stabilized atomically precise AuNCs can be viewed as multi-shell systems composed of a gold core (leading to core states), a gold–ligand interface (mainly Au-S bonding leading to “surface” states), and a ligand shell. The three shells may communicate, leading to subtle charge transfer processes. Transitions from molecular orbitals with strong ligand contributions to orbitals with high metal characters (LMCT) and from metal-to-metal electronic transitions can cause near-ultraviolet and visible absorption. Clusters with a core of metal atoms have an initial decay pathway that could result in emission in the visible spectrum. All clusters have a charge-transfer component with a long-lived decay [87]. Furthermore, intersystem crossings are associated with multiple energy transfers (reinforced intersystem crossing (ISC)), which may result in an overall increase in photoluminescence (PL) emissions and longer PL lifetimes [79]. The luminescence property is affected by temperature, in such a way that, upon decreasing the temperature, the HOMO-LUMO gap increases [46].

#### 1.4.3. Two-Photon Absorption

**Figure 6:**
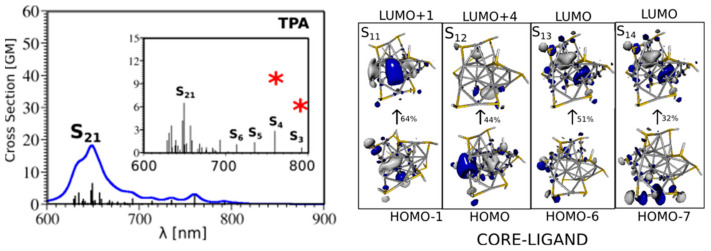
TDDFT TPA spectra of Ag_15_(SR)_11_ nanoclusters for the lowest-energy structure (**left panel**). Leading excitations responsible for the large TPA cross-sections illustrating the participation of the ligands and the core are also shown (**right panel**).

Two-photon-absorbing (TPA) photocatalysts use near-infrared low-energy photons [88] for photocatalysis. The unique, nonlinear optical (NLO) properties of sub-nanometer core-sized clusters of MNCs exhibit outstanding characteristics [21]. Compared to conventional organic dyes, MNCs show superior two-photon absorption cross-sections (TPA). Although these two-photon processes in small, numbered MNCs have been well established, the basic photophysical mechanisms behind them still need to be better understood. Generalizations based on complementary theoretical and experimental studies have been made possible by their two-photon absorption properties [89]. It has been reported that the TPA characteristics of AuNCs and AgNCs can be enhanced by the concept of a “ligand-core NLO-phone” [90,91]. The different electron or charge transfer processes between the metallic core and ligand are essential for boosting the transition dipole moments, leading to enhanced TPA cross-sections (as exemplified in Figure 6 with Ag_15_(SH)_11_ nanoclusters).

#### 1.4.4. Chirality

**Figure 7:**
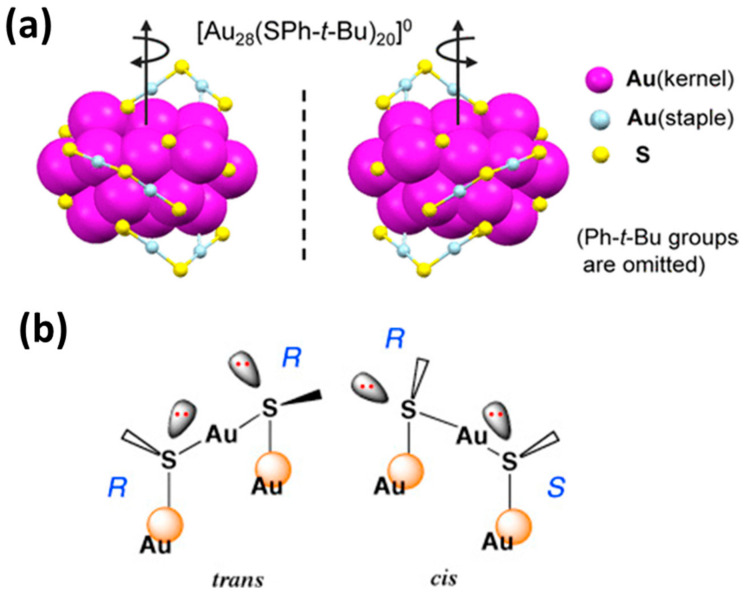
(**a**) The two enantiomers of Au_28_(TBBT)_2_^0^, where TBBT = 4-tert-butylbenzenethiolate, exhibiting a rod-like Au_20_ kernel consisting of two interpenetrating cuboctahedra. (**b**) Structures of the (R, R)-trans (left) and (R, S)-cis (right) configurations of two thiolate ligands. Reprinted with permission from [40,94]. Copyright 2016 Elsevier.

The photocatalytic activity induced by chiral metallic particles varies with the helicity of the illumination light [92]. MNCs such as gold quantum clusters display fascinating chiral properties [93]. Moreover, this chirality depends mainly on three factors [46]; (a) fundamental chirality, which is induced first by the atomic packing mode, (b) the geometrical isomerisms of the surrounding motifs, and (c) the natural chiral characteristics of the protective ligands and their arrangements covering the metal core [51]. Figure 7a illustrates the two enantiomers of Au_28_(TBBT)_20_, where the origin of chirality is primarily rooted in the rotating arrangement of the four dimeric staples, as well as the arrangement of the bridging thiolates (quasi-D2 symmetry) [40]. It is worth noting that the SR-Au-SR units of AuNCs display chirality, a chiral center at each sulfur atom, and can exist in either an (R, R)/(S, S) trans-configuration or an (R, S) cis-configuration. Indeed, the electronic structure of the sulfur atom in the SR-Au-SR units can be regarded as the sp3 type, where electrons participate in four tetrahedral kinds of interactions [94]. This bonding motif then creates a chiral center at the sulfur (Figure 7b).

### 1.5. Stability

Metal clusters shielded by thiolates or polymers are attractive possibilities for nanoscale devices [95]. For instance, among the various GSH-protected AuNCs, Au_25_SR_18_ is the most stable one [96]. Isolated Au_55_ clusters exhibit an amazing resistance to oxidation, even when exposed to the oxygen atoms and radicals produced by oxygen plasma [97]. When the particle size is decreased to 1.6 nm, the metallic component’s spectral weight increases rapidly, indicating that these particles are more oxidation-resistant. This resistance is much greater for the case of naked Au_55_ clusters. In addition, ligands influence the stability of MNCs such that the thermal stability of captopril-protected Au_25_ is greater when compared to GSH-protected AuNCs [98].

Doping the central metallic core of Au_25_ with other elements such as Cu and Pd alters its geometric structure and increases its resistance to deterioration, respectively [99]. To illustrate this, Negishi, Y., Kurashige, and co-workers [100] doped Au_25_(SG)_18_ with Pd, leading to the formation of Pd_1_Au_24_(SC_12_H_25_)_18_. [Pd_1_@Au_24_(SC_12_H_25_)_18_] is a binary Pd Au core–shell nanocluster, in which Pd is positioned at the center of the icosahedral core of the nanocluster. This was prepared by replacing the central Au atom of [Au_24_(SC_12_H_25_)_18_] with Pd, and the resulting binary mixture exhibited an increased thermodynamic stability. This stability against degradation was analyzed by monitoring the absorption spectra of a toluene solution containing [Au_24_(SC_12_H_25_)_18_] and [Pd_1_@Au_24_(SC_12_H_25_)_18_]. They found that [Au_24_(SC_12_H_25_)_18_] was stable for up to 30 days in an organic reactor containing toluene as a solvent. Thus, they noticed that Pd-doped AuNCs exhibited an increased thermodynamic stability and stability against degradation. Similar trends have been observed with Pt-doped metal nanoclusters [101].

The spectral profile evolves with time, and after 30 days, it resembles that of Pd_1_Au_24_(SC_12_H_25_)_18_ rather closely and the ESI MS spectrum shows just a single peak, which may be ascribed to Pd_1_Au_24_(SC_12_H_25_)_18_ [102]. The strong interaction energy between Pd and Au_24_(SC_12_H_25_)_18_ was proved by the DFT calculation of Jiang et al. Consequently, with the Au_24_(SC_12_H_25_)_18_ frame, Pd develops an intermetallic structure and, with the strength of this, Pd_1_Au_24_(SC_12_H_25_)_18_ presents a higher thermodynamic stability than Au_25_(SC_12_H_25_)_18_. On the other hand, doping Au_25_(SC_2_H_4_Ph)_18_ with copper reduces its overall nanocluster stability [103]. Doping with copper forms Cu_~1_Au_~24_(SC_2_H_4_Ph)_18_. In comparison to Au_25_(SC_2_H_4_Ph)_18_, the optical absorption spectra moves toward lower energy, suggesting that the HOMO-LUMO gap shrinks.

**Figure 8:**
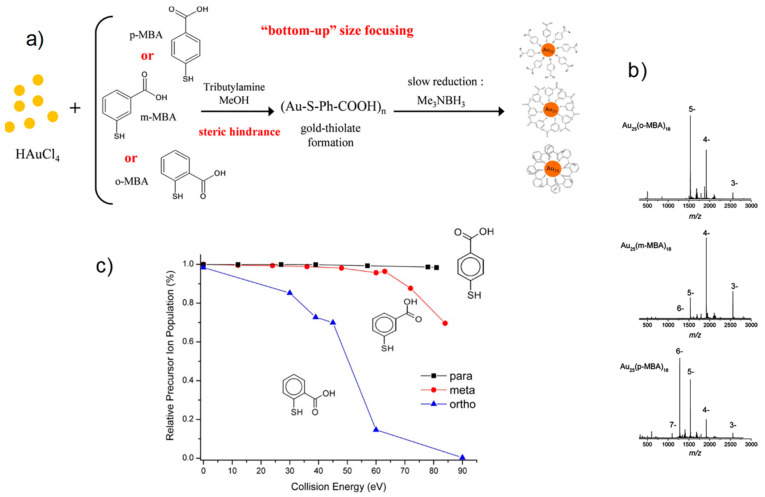
(**a**) Diagram shows the synthesis protocol of different structural MBA-isomer-stabilized Au_25_(MBA)_18_ NCs. (**b**) ESI mass spectra Au_25_ clusters, and (**c**) collision-induced dissociation breakdown curves for the 4– charge state of o/m/p-MBA stabilized. Reprinted with permission from [106] Copyright 2018 American Chemical Society.

Studying the electronic densities of the states of Au_25_(SR)_18_^1−^ reveals the electronic state of its Au_13_ core (eight electrons), protected by six [(SR)_3_Au_2_] complexes [68]. The superior stability of Au_25_(RS)_18_ is achieved by structural toughness and an eight e- shell of delocalized Au(6s) electrons for the anion. The gold nanocluster superatom model expands the jellium model [104] to explain multiple ligand-protected AuNCs with closed valence-electron shells (2, 8, 18, 20, 34, and 40, etc.). The metal core is regarded as a single atom in the super atom hypothesis. The extraordinary stability of these NCs emerges from the regular closure of outer electronic shells [105]. Even if a strong structural and electronic stabilization occurs for Au_25_(RS)_18_, the nature of the protecting ligands affects the stability of the cluster. The role of the isomer was studied using para, meta, and ortho MBA-stabilized NCs [106]. Figure 8 shows the isomeric effect of MBA on the stability of Au_25_(MBA)_18_ NCs. The steric hindrance of the carboxylic groups plays a major role. The m-MBA- and o-MBA-ligand-stabilized nanocluster shows a noticeable steric hindrance at the gold core’s surface, which leads to a significant lowering of the binding energy required for the fragmentation of the Au-S bond.

## 2. Application of Nanoclusters in Photocatalysis

Over time, research on the relevance of cleaner fuels and alternate potential energy sources, as well as tools for alleviating pollution, have been gainful. Research on visible light photocatalysis over the past decade has drawn much attention in this line. Semiconductor heterogeneous nanotechnology-based photocatalysts have lasted long and showcased their prominence in this specialty. At present, material chemists are busy preparing competent monometallic or hybrid entities by combining conventional semiconductors with suitable pre-catalyst/co-catalysts, in order to bring about the best results in photocatalysis. MNCs have proved their supremacy in multiple economic and sustainable photocatalysis processes, such as the photodegradation of organic pollutants, photocatalytic H_2_ splitting, the photoreduction of CO_2_, and oxidation and hydrogenation reactions, etc. The fast recombination of the e^−^/h^+^ of semiconductors and the noble metal/metallic/non-metallic moieties on semiconductors support extending photocatalytic activity towards the visible range.

Photostability and recyclability are considered to be major factors to consider during photocatalysis. The photocatalytic stability of NCs has been improved by incorporating a suitable catalyst, which further enhances the efficiency of the catalysis [107,108]. Duan and his co-workers developed TiO_2_-supported AgNCs for the photocatalytic removal of NO. They studied the photostability of the TiO_2_-NCs, which showed excellent photostability and recyclability [109]. A similar observation was observed by Yu et al., where they found that TiO_2_ nanocrystals-supported Au_25_(SR)_18_ shows an outstanding photostability in cycle studies and an enhanced photoactivity for methyl orange (MO) degradation [110].

The following section discusses the role of MNCs as cocatalysts, as well as catalysts in various light conversion processes.

### 2.1. Photodegradation of Organic Pollutants

Globalization-led industrialization, in order to satisfy the needs of the world’s population, has led to a steep rise in harmful pollutants in the environment at an alarming rate. The organic pollutants from textile industries majorly constitute azo dyes, while inorganic pollutants hold oxidized heavy metals. These under-treated materials containing carcinogenic effluents are being dumped into soil and water and have already created damage beyond repair to humans, as well as to aquatic flora and fauna. Initiating action plans to safely remove them without the formation of other stable secondary pollutants demands scrutiny. Methyl orange (MO), rhodamine B (RB), malachite green (MG), and methylene blue (MB) are some of the commonly found dyes that are present in industrial effluents. Reactive oxygen species (ROS), such as superoxide radicals (O^2−.^) and hydroxyl radicals (OH^−.^), are the prime contributors and starting materials in the degradation of these toxic materials [111].

Titania (TiO_2_), ZnO, SiO_2_, and Nb_2_O_5,_ etc., have been widely reported as a solution for pollution, initiated by organic pollutants through photodegradation [112,113]. These semiconductor systems ensure photostability, a low cost, mere toxicity, and a fundamental level of oxidative ability. However, the fast recombination of electron-hole pairs before taking part in surface reactions reduces the rate of the photoreactions. Grafting small nanoparticles onto TiO_2_, which can act as a co-catalyst to efficiently absorb in the visible and possibly NIR range, acts as an electron acceptor, and, in turn, suppresses the recombination of photo-excited electron−hole pairs, which would dramatically enhance their efficiency. As research studies have progressed, metal-nanoparticle-incorporated versions of semiconductors have become the focal point. MNCs linked to semiconductor systems for support have profoundly influenced works on the photocatalytic degradation of cationic and anionic dye pollutants, as they could enable us to engineer the bandgap width [114,115].

Zhu and co-workers prepared AuNCs coupled with toroid-structured per-6-thio-β-cyclodextrins placed on the TiO_2_ surface (TiO_2_-Au NCs@β-CD), providing a better space for the Au cores to interact with the incoming pollutants through a host–guest interaction trap [116]. This synergistic effect between the nanocluster metallic core, peculiar ligand cavities, and support system increased the photodegradation rate of methyl orange (MO) to 98% in 10 min. Concurrently, the integrated material’s rate constant values for the photodegradation (first cycle = 0.31 min^−1^, fifth cycle = 0.15 min^−1^) were phenomenal in comparison to TiO_2_ (first cycle = 0.12 min^−1^), even after five cycles. Sharma and co-workers studied a Au-TiO_2_-conjugated nano-assembly as a photocatalyst under both UV and visible light by utilizing methylene blue and a common organic pollutant carbendazim [117].

The mechanism for the photocatalytic degradation of dyes using Au-TiO_2_ can be explained by the following reaction pathways:

Au + hν → Au•

Au• + TiO_2_ → h+(Au) + e-(TiO_2_)

e-(TiO_2_) + O_2_ → TiO_2_ + •O^2−^

•O_2−_ + H^+^ → HOO•

HOO• + e-(TiO_2_) + H^+^ → H_2_O_2_ + TiO_2_

H_2_O_2_ + e-(TiO_2_) → •OH + OH^−^ + TiO_2_

h+(Au) + H_2_O → Au + H^+^ + •OH

h+(Au) + OH^−^ → Au + • OH^−^

Organic pollutants + O_2−_• or •OH^−^ → CO_2_ + H_2_O +…

**Figure 9:**
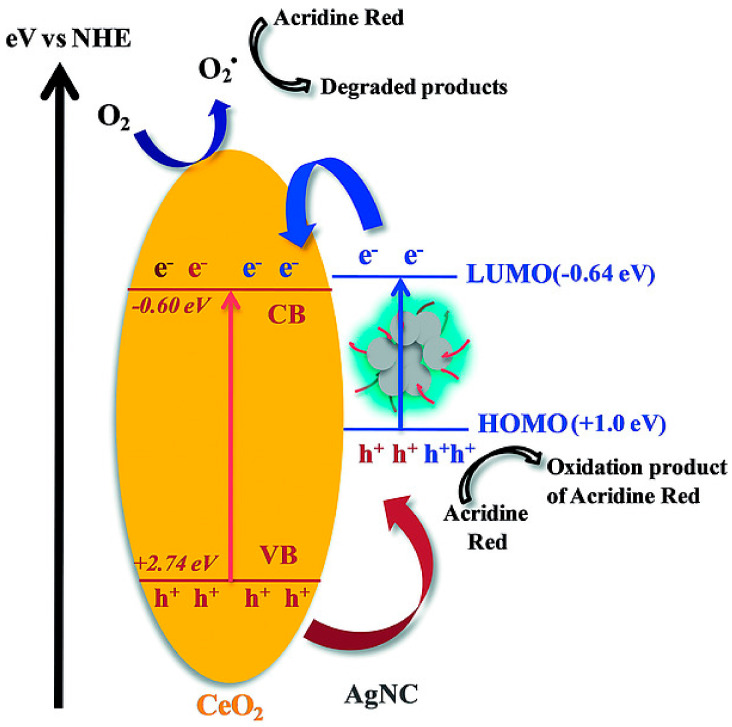
Schematic representation of the mechanism of the photocatalytic degradation of Acridine Red in the presence of the AgNC/CeO_2_ nanocomposite under UV light irradiation. Reprinted with permission from [119] Copyright © 2018 John Wiley & Sons.

Gowswami et al. created a colloidal nanocomposite material with TiO_2_/Nb_2_O_5_ conjugated to silver NCs and captopril as a ligand. They varied the niobium loading in the incorporated product to investigate the adhesion of the material towards cationic and anionic dyes, as well as their photodegradation capability [118]. As per the analysis, the mentioned nanocomposite with 48.1% niobium loading and Ag NCs resulted in a ternary junction, which narrowed down the recombination rates and surface acidity caused the Ag NC ligands’ end groups to polarize COO^−^, thereby attracting the cationic dyes for an effective 100% degradation with a high photostability. Spectroscopic techniques validated that the Ag NCs were the reason behind the absorption in the visible light region and acted as oxidative sites. They also initiated electron transfer from the valence band of the NCs to the conduction band of the nanocomposites. Samai’s group made polyethylene imine template Ag NCs incorporated with CeO_2_ nanoparticles to photo-catalytically degrade acridin red dye [119]. The XPS mechanistic work and radical experiments pinned down their radical pathway, leading to between 42% and 80% degradation in 2 h, with 1.07% and 3.10% Ag NCs loading, respectively. The reduction potential and two oxidation potentials vs. the NHE values were recorded with a cyclic voltammogram to be −0.64 V, 0.07 V, and 1.03 V, respectively (Figure 9).

**Figure 10:**
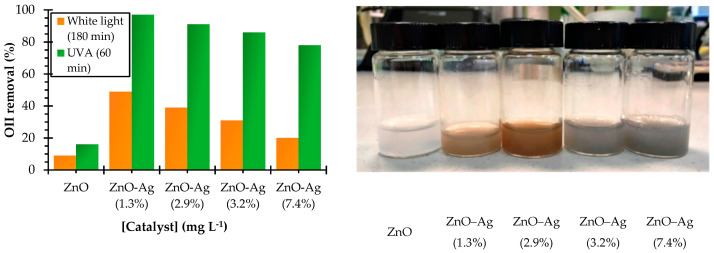
Silver loading effect on photocatalytic performance. The values in brackets correspond to the percentage of Ag in each NC (**left**); Aqueous suspensions of ZnO-NPs and ZnO–Ag NCs with different silver loadings (**right**). Reprinted with permission from [120] Copyright 2019 MDPI.

ZnO nanoparticles decorated with Ag NCs were established as prospective candidates for Orange II (OII) dye degradation under both UV and white light by Rodriguez and co-workers [120]. Atomic force microscopy (AFM) confirmed the deposition of the NCs’ moiety on ZnO, rather than a substitution of the metallic core itself. Additionally, the optimal loading range of the Ag on ZnO was found to be 1.3% *w*/*w* and it indeed ensured an ample number of interaction sites for the pollutants to approach the ZnO nanoparticles. Figure 10 shows the spectroscopic and morphological characterization of these AgNC-decorated ZnO nanoparticles. Vidal et al. formulated green-emitting stable closed-shell electronic structured and recyclable CuNCs to degrade MB under UV and visible light irradiation [121]. The UV-Vis data and administered multiple photoluminescence emission peaks were an indication of a mixture of clusters and LDI-TOF spectrometry approved the proposed structural formulas of the Cu NCs: [Cu_18_(CH_3_COO)(OH)]^−2^ and [Cu_34_O_2_(CH_3_COO)_3_N(C_4_H_9_)_3_Na]^−2^.

A light-steered preparation of 3-mercaptopropyl trimethoxy silane (MPTS) stabilized AuNCs, and their performance in the photodegradation of MB was examined by Zhou et al. [49]. It was observed that the NCs progressively decolorized the dye, and the color faded completely in 60 min.

Man Cao and his co-worker synthesized a self-assembled silver nanocluster for the photocatalytic degradation of a sulfur mustard simulant (2-chloroethyl ethyl sulfide, CEES), a toxic vesicant against human proteins and DNA that can cause skin blisters, eye and respiratory system irritation, and even fatal damage. The silver cluster assembled material was prepared using a photosensitizer and 5,10,15,20-tetra(4-pyridyl)porphyrin (TPyP) as the organic linker, which linked with 12-core silver chalcogenolate cluster to form [Ag_12_(St Bu)_6_(CF_3_COO)_3_(TPyP)]_n_, (designated as Ag_12_TPyP). They reported a 98% degradation of CEES with 1% loading [122,123].

Wen and co-workers developed a highly stable core–shell-type catalyst for photo-redox reactions. The photostability of the nanocluster was improved by loading it onto a SiO_2_ sphere by utilizing multifunctional branched poly-ethylenimine (BPEI) as a surface-charge-modifying, reducing, and stabilizing agent. Then, TiO_2_ was coated with SiO_2_-Au GSH--BPEI to form a SiO_2_-AuGSH--BPEI@TiO_2_ core–shell structure, significantly improving further the photocatalytic efficiency for the degradation of organic dye, Rh B [124].

### 2.2. Oxidation and Hydrogenation Processes

**Figure 11:**
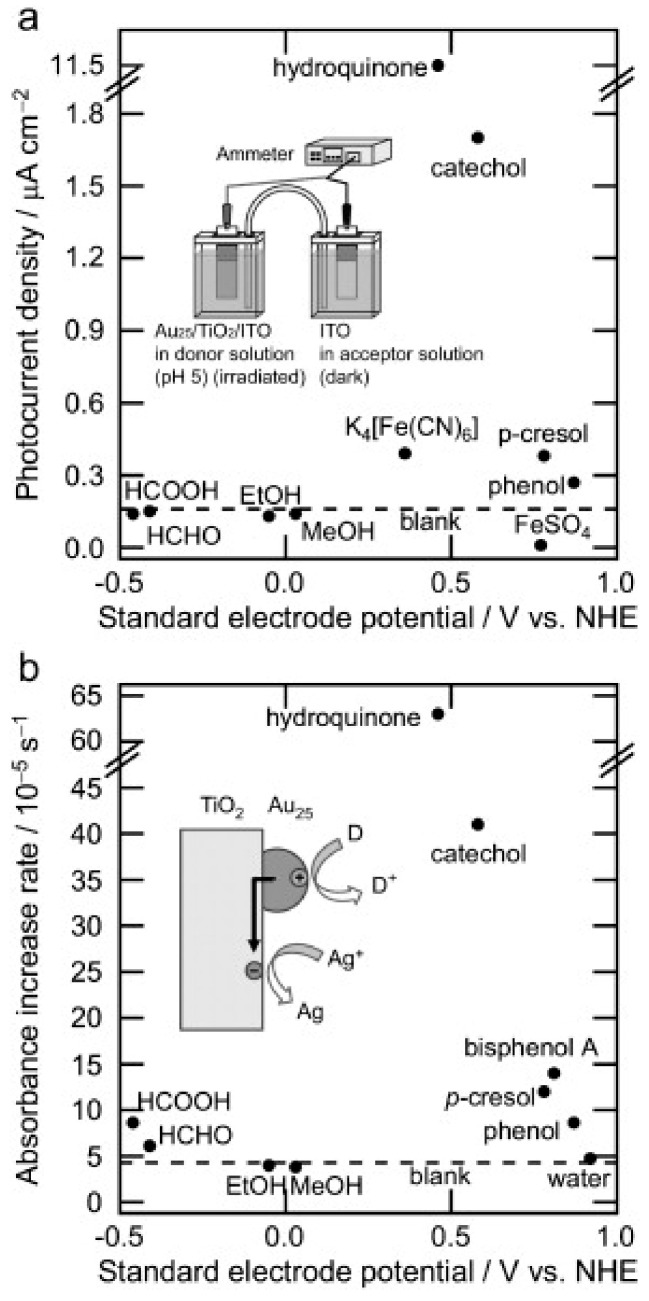
Photocatalytic oxidation ability of the Au_25_-TiO_2_ under visible light irradiation accompanied by reduction of Ag^+^: (**a**) shortcircuit photocurrent densities measured in the two-compartment cell (inset) and (**b**) absorbance increase rates plotted against E° of the donors. Reprinted with permission from [129] Copyright 2010 Elsevier.

The application of NCs in oxidation and reduction processes is still underway. Oxidation and hydrogenation reactions, in most reported cases, specifically depend on their electronic structures, as electron-hole separation remains the key to this. Researchers have carried out typical oxidation reduction reactions such as high-selectivity styrene oxidation [125], the aerobic oxidation of amines to imines [126], and cyclohexane or phenol derivatives [127] in the presence of MNCs, especially AuNCs. In 1987, Haruta and co-workers were the first to initiate the oxidation of CO with a few atom AuNCs, along with α-Fe_2_O_3_, NiO, and Co_3_O_4_ at low temperatures [128]. After two decades, a mechanistic view of the photocatalysis of Au_25_ nanoclusters with TiO_2_ as a support under visible and near-infrared emissions was illustrated by Kogo and co-workers [129]. They attached AuNCs to TiO_2_ so that the excited electrons could be transferred to the conduction band of the TiO_2_ with ease and aid in the reduction of Ag^+^ in the counter electrode, and the generated holes (h^+^) could perform the oxidation of the donors (phenol derivatives or formic acid) in the working electrode (Figure 11).

Similarly, Zhu et al. performed a comparative study to figure out the capabilities of a set of superatoms for oxidizing styrene Au_25_(SR)_18_, Au_38_(SR)_24_, and Au_144_(SR)_60_ with diameters of 1 nm, 1.3 nm, and 1.6 nm, respectively [130]. However, their studies reinforced the size and electronic structure dependence of the photocatalytic abilities, and Au_25_ NCs gave the highest average of ~27 ± 1.0%, as the smaller the superatom, the higher the HOMO-LUMO gap (1.3 eV). Later, Chen and their group loaded [Au_25_(PPh_3_)_10_(SR)_5_Cl_2_] on P_25_ to convert benzylamines to imines; the TOF was recorded to be 1522 h^−1^ for 4-methylbenzene and other amines also showed appreciable conversions (73–99%) [126]. The attempts to learn the possible conversion route using time-dependent density functional theory (TD-DFT) calculations, Fourier-transform ion cyclotron resonance mass spectrometry (FT-IC-MS) with electrospray ionization (ESI), and trapping intermediates with scavengers (K_2_S_2_O_8_ and ammonium oxalate) confirmed the presence of a +2 charge on the cluster. Hence, the photo-catalytically-induced electron could lead to oxygen radical formation and thereby persuade conversions. Studies with organic support have also gained momentum over the years. Gold nanoclusters with organic supports (polyvinylpyrrolidone (PVP) and polyperoxyacetic acid (PAA)) were analyzed by H.Tsunoyama and co-workers. X-ray photoelectron spectroscopy (XPS) and X-ray absorption near edge structure (XANES) reinforced the existence of negatively charged clusters, which led to the production of the superoxide- or peroxy-radicals required to jump-start the photocatalytic reactions [131]. Their activities and recyclability were keenly observed for CO oxidation. In addition, this set of reactions enlightened the research community about the prime role of NCs in oxidation and reduction reactions.

Hamoud and co-workers studied the photocatalytic activity of Ag NCs encapsulated into zeolite (ZX-Bi zeolite) for the photooxidation of methanol under visible light. They found that the Ag/ZX-Bi exhibited very low activity compared to the activated sample at 200 °C (Ag/ZX-Bi_200) [132].

### 2.3. Photocatalytic H_2_ Production

#### 2.3.1. Hydrogen Evolution Reaction

The demand for green energy sources better suited for the environment is rising due to the reductions and environmental pollution generated by conventional energy resources. Hydrogen gas is a suitable renewable energy source for a sustainable future, even though its distinctive properties need to be refined. [133]. Ahluwalia and co-workers studied the fuel economy of hydrogen-fueled fuel cell (H_2_-FCV) vehicles and common gasoline-fueled passenger cars in 2003 [134]. In 1972, Fujishima et al. first discovered that water could be divided into H_2_ and O_2_ in the presence of light [1].

Catalysts based on metals such as Ti, Cobalt, Nickel, Iron, and Molybdenum are widely used as catalysts for electrochemical H_2_ production [135]. After this, the catalytic efficiency of metal oxides and metal nitrides was enhanced by incorporating suitable cocatalysts into these semiconducting materials. The co-catalytic activity of metal nanoclusters has been studied in the recent past due to their excellent optical, electronic, and catalytic activity. NCs have been incorporated into various semiconductors to enhance their catalytic activity by suppressing the electron-hole recombination rate.

Kamat et al. studied the photoelectrochemical and photocatalytic production of H_2_ using a GSH-stabilized AuNCs-TiO_2_ film hybrid system under visible light irradiation [135]. Liu et al. carried out a detailed investigation on the photocatalytic activity of AuNCs as cocatalysts in highly ordered nanoporous layer-covered TiO_2_ nanotube arrays (NP-TNTAs). The photocatalytic activity of the NP-TNTAs/AuNCs was analyzed by monitoring the photodegradation of organic dyes, the photocatalytic reduction of aromatic nitro compounds, and photoelectrochemical water splitting [136]. Similarly, AuNCs loaded on SrTiO_3_ were studied for the HER and an enhanced catalytic activity of SrTiO_3_ was found in the presence AuNCs as cocatalysts [137].

The quantum confinement of MNCs enables the charge transfer, easy adsorption, and desorption of intermediates, thereby fastening the photocatalytic hydrogen evolution reactions (HER) [29]. Sub-nanosized clusters of silver adsorbed on specific sites of gold nanorods (GNRs) disturb the growth symmetry of Au facets and lead to anisotropy [138]. In the presence of a hole scavenger, namely ethanol, and in the absence of electron scavengers such as O_2_, the photoelectrons accumulate in GNRs with Ag clusters. Moreover, under low UV light irradiation, Ag_3_ clusters at a concentration of 0.43µg within GNRs show a high H_2_ production efficiency of 10%. Similarly, the loading of 1 wt% of sub nm Au clusters on CdS uplifts the photocatalytic H_2_ production by about 35 times that of the unmodified CdS under visible light [139]. The comparison of sub nm Au-loaded CdS with Pd/CdS and Rh/CdS of similar sizes reveals that 3 nm sized Au/CdS is a better co-catalyst.

Likewise, monolayer niobate (HTi_2_NbO_7_) nanosheets with Pt NCs have proved themselves as potential photocatalysts for a high H_2_ production [140]. Monolayer niobate nanosheets enable the charge separation between photoelectrons and holes and display a five times higher photocatalytic H_2_ production than that observed in its layered form. 1 wt% Pt NCs loaded on niobate nanosheets via photoreduction present increased activity due to the close contact between the HTi_2_NbO_7_ nanosheets and Pt nanocluster. Under light irradiation, electrons migrate to the conduction band moved to the surface of the nanosheets. Since Pt has a higher work function than niobate nanosheets, these electrons on the surface of nanosheets transfer to Pt and form H_2_.

Cheng and co-workers [138] revealed the effective co-catalytic effect of sub-nano-sized Pt-Au alloy clusters in a photocatalytic H_2_ evolution. The synergistic effect of 0.5 wt% of both Pt and Au clusters dispersed in TiO_2_ (Pt-Au/T) created an increased charge separation, and 80.1 µmol h^−1^ of H_2_ was evolved with a quantum efficiency of 50% at 365 nm. As with the HER, a similar strategy has been established for an enhanced efficiency of the OER by using the nanocluster as a cocatalyst [141]. A Au_25_ NC- CoSe_2_ composite was studied for its OER activity and enhanced OER activity was found in the presence of Au_25_/CoSe_2_, obtaining a current density of 10 mA cm^−2^ at a small overpotential of ∼0.43 V (cf. CoSe_2_: ∼0.52 V). The ligand and gold cluster size could also tune the catalytic performances of the composites. Yang et al. also illustrated the effect of heteroatom doping on the photocatalytic activity of PtAg_24_-loaded graphitic carbon nitride (PtAg_24_/g-C_3_N_4_) and found that PtAg_24_/g-C_3_N_4_ showed a higher efficiency for photocatalytic H_2_ production than Ag_25_/g-C_3_N_4_ alone [142].

Similar to Au, Ag, Pt, and Pd NCs, Cu nanoclusters are also used as photocatalysts for various photoreactions. Barbara et al. developed in situ formations of CuNCs over hexaniobate nanosheets for a photocatalytic H_2_ evolution reaction. The electrostatic interactions between Cu and Ni led to the decoration of the NCs over the hexaniobate nanosheets, increasing the electron-hole separation and thus inducing an enhanced efficiency for H_2_ evolution [121,143,144,145].

#### 2.3.2. Photocatalytic Water Splitting

SG-stabilized AuNCs (Au_25_(SG)_18_) have been incorporated on BaLa_4_Ti_4_O_15_ for photocatalytic water splitting. The catalytic activity of sub-nanometer-sized Au-BaLa4Ti4O_15_, in comparison larger-sized gold nanoparticles, found a 2.6 times higher catalytic activity for the AuNC composite [146]. Recently, Hanieh Mousavi and co-workers studied the photocatalytic production of H_2_ using AuNCs as co-catalysts. They prepared a nanocomposite (Au_101_NC-AlSrTiO_3_-rGO) containing AuNCs, RGO, and AlSrTiO_3,_ and the composite showed enhanced photocatalytic water splitting under UV light irradiation [147].

Moreover, by monitoring the nanocluster size, the photocatalytic activity of the whole system can be varied. Heiz, Feldmann, and co-workers modified cadmium sulfide (CdS) nanorods with a series of Pt NCs, such as Pt_8_NC, Pt_22_NC, Pt_34_NC, Pt_46_NC, and Pt_68_NC, and found that Pt_46_/CdS exhibited the highest activity for photocatalytic water splitting, due to the well-known quantum confinement effect, where the bandgap increases with a decrease in the NC size [148]. Rongchao and his co-workers reported a detailed review of the effect of the NCs’ size on photocatalysis [85].

The photocatalytic activity of co-catalysts have been studied by monitoring the doping of a heteroatom on NCs. Negishi et al. demonstrated that doping Pt on Au_25_ NC enhanced the water-splitting activity, while Pd doping reduced this water-splitting activity. They proposed that the doping position plays a role in the catalytic activity. The doped Pd was located at the surface of the metal cluster cocatalyst, whereas the Pt was located at the interface between the metal cluster cocatalyst and the photocatalyst [149]. In addition to the above studies, scientists are exploring the efficiency of photocatalytic water using composites, including MNCs with g-C_3_N_4_ nanosheets and TiO_2_, etc. [150].

### 2.4. Photocatalytic CO_2_ Reduction

Today, reducing the greenhouse gas emissions from various photovoltaic (PV) systems has become one of the scientific community’s primary concerns [151]. Carbon dioxide (CO_2_) is one of the chief greenhouse gases that influences the heat content of the Earth’s atmosphere [1]. With a focus on reducing CO_2_ emissions, novel technologies have been adopted for the production of commodity chemicals by using CO_2_ as feedstock [152]. The conversion of CO_2_ to value-added chemicals or other hydrocarbon fuels, such as methane, ethylene, and carbon monoxide, by utilizing energy from non-fossil resources such as solar energy increases carbon recycling and assists in fuel production [153,154]. The solar-driven transformation of CO_2_ into valuable products could be achieved through two major approaches, such as photocatalytic and electrochemical CO_2_ reduction processes [155]. In 1978, Halmann utilized p-type semiconductors for the photo-electrochemical reduction of CO_2_ [156]. Methanol and carbon monoxide obtained from the conversion of CO_2_ have been identified as useful feedstocks [157]. The former synthesizes other hydrocarbon fuels, while the latter is used for Fischer–Tropsch syntheses.

CO_2_ is a thermodynamically stable molecule, and catalysts assist in electrochemical CO_2_ reduction reactions and aid in achieving the desired product [158]. MNCs possess ultrafine structures, electronic and optical properties [34], and function as electrocatalysts and photocatalysts [27]. Various features of MNCs, such as their size, core, composition, surface ligands, charge state, and geometry, influence their electro and photocatalytic behavior [27]. Colombo Jr and co-workers studied a femtosecond electron-hole recombination in TiO_2_-NCs and explained the intra-cluster dynamics [159]. The study demonstrated the steps involved in electron trapping, recombination, and the formation of long-lived species. Kauffman et al. studied the weak reversible interaction between CO_2_ and Au_25_(SC_2_H_4_Ph)_18_^−^ clusters [160]. The electrochemical reduction of CO_2_ using a Au_25_ catalyst in aqueous 0.1 M KHCO_3_ showed a maximum CO production at −1.0 V with a 100% Faradaic efficiency. The electrochemical CO_2_ reduction performance of silver NCs confined in bovine serum albumin (AgNC@BSA) is enhanced via polyoxometalates [α-SiW_12_O_40_]^4−^ [161].

The presence of suitable photocatalysts possessing features such as a high light absorptive power, convenient catalytic sites, and a low activation energy enhances the photocatalytic reduction of CO_2_ [162]. The small-scale size of MNCs of about 2 nm, interfacial surfaces, energy gaps, tunable chemical properties, and quantum confinement are the advantages of ultrafine MNCs for CO_2_ reductions over metal nanoparticles [163]. Titanium dioxides or titania are widely used semiconductors and have a broad range of applications, including the photoreduction of CO_2_ [164]. Doping and decoration with other elements or metal ions strengthen their photocatalytic activity by reducing the band gap. In addition to these elements, attaching NCs to TiO_2_ makes it a potent visible light photocatalyst [165]. Inserting Ti^3+^ ions into TiO_2_ creates isolated states in the presence of UV and visible light. The electrons are trapped in these states, and due to recombination with charge carriers, the photocatalytic activity is decreased. Upon combining Ti^3+^-introduced TiO_2_ with NCs of Cu (II) oxides (i.e., Cu (II)-TiO_2_@Ti^3+^), electrons from the isolated states of Ti^3+^ move to the surface of the Cu (II) NCs. Hence, the photocatalytic activity of Cu (II)-TiO_2_@Ti^3+^ under visible light is raised and gaseous 2-propanol (IPA) is decomposed completely to yield a ~18 µmol CO_2_ generation. Under UV irradiation, copper oxide (Cu_x_O) NCs incorporated into strontium titanate nanorod thin films [166] and niobate sheets [144] show a selective CO production from photocatalytic CO_2_ reduction.

**Figure 12:**
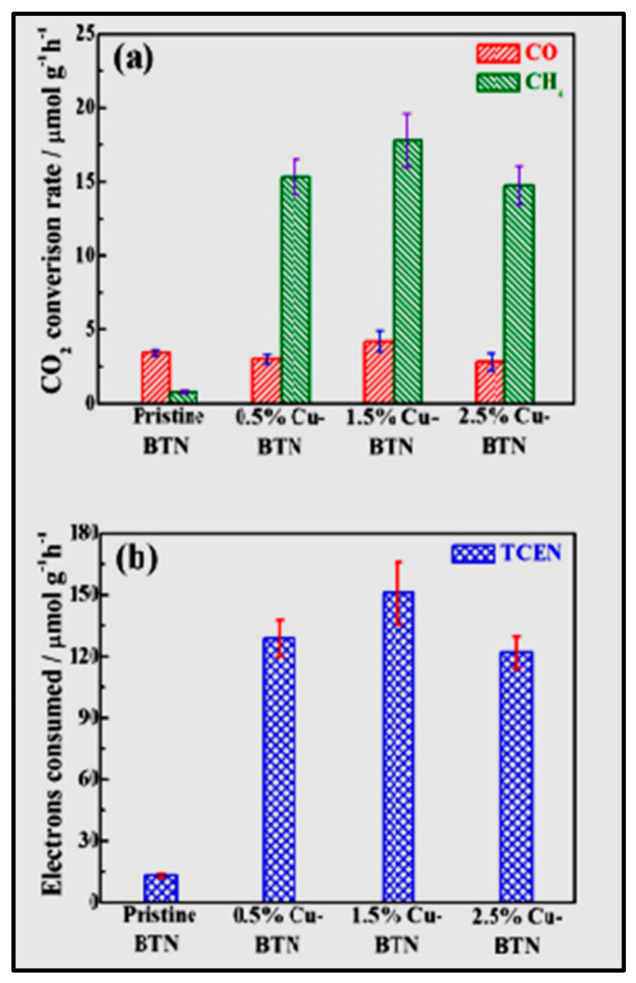
CO/CH_4_ production rates (**a**), and the corresponding total consumed electron numbers (TCEN) (**b**) for the CO_2_ photoreduction over pristine BTN and Cu-BTN production with different Cu loading contents during the initial 2 h of irradiation. Reprinted with permission from [167] Copyright 2017 American Chemical Society.

The product selectivity towards CH_4_ and CO is shown by brookite TiO_2_ quasi nanocubes (BTN) on surface decoration with Cu-NCs (Cu-BTN) under xenon lamp irradiation [167]. XRD diffraction peaks exhibit the presence of Cu-NCs, only on the surface of BTN. The total consumed electron number (TCEN) is utilized for examining the overall photocatalytic CO_2_ reduction. At a 1.5% optimum concentration of Cu-NCs in BTN, a maximum photoactivity with TCEN of 150.9 µmolg^−1^ h^−1^ and highest production rate of 4.23 µmolg^−1^ h^−1^ CO and 17.81 µmolg^−1^ h^−1^ CH_4_ are observed (Figure 12).

**Figure 13:**
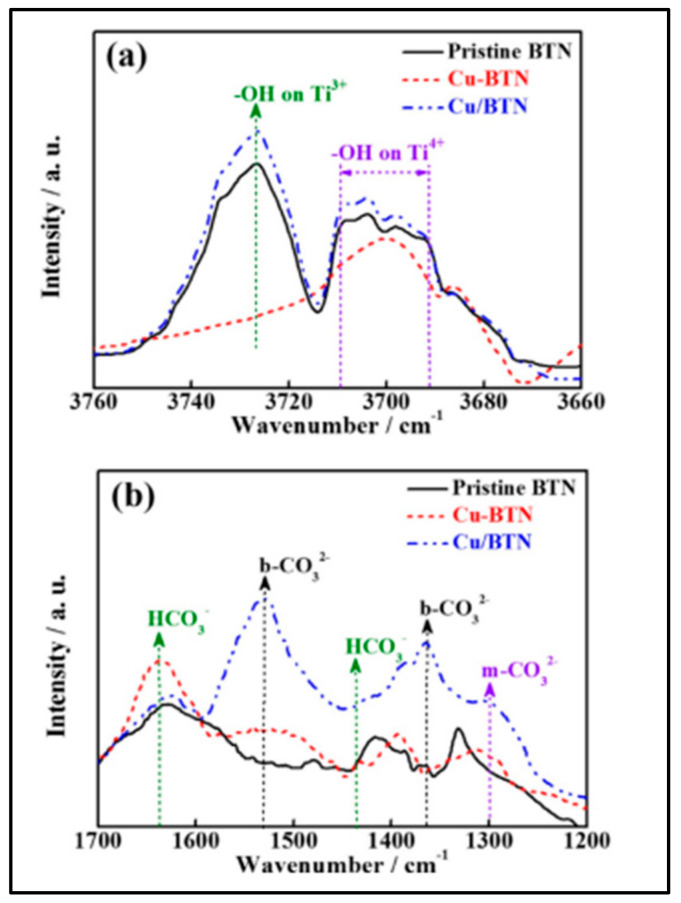
In situ DRIFTS IR spectra of H_2_O (**a**), or CO_2_/H_2_O vapor (**b**) interaction with pristine BTN, 1.5% Cu-BTN, and 0.5% Cu/BTN. Reprinted with permission from [167] Copyright 2017 American Chemical Society.

**Figure 14:**
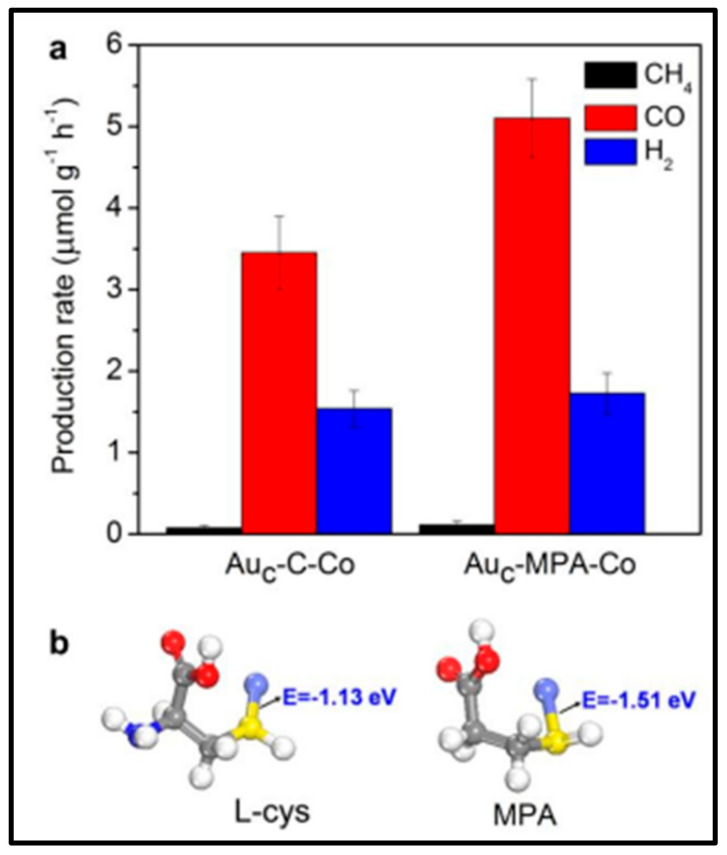
(**a**) Average production rates of CH_4_, CO, and H_2_ in light-driven CO_2_ reduction with H_2_O in the presence of TEOA, catalyzed by 10-mg Au_c_-MPA-Co grafted with 4 µmol Co^2+^ (**b**) The binding energy of S-Co bond in the coordination of Co with L-cys and MPA. Reprinted with permission from [168] Copyright 2018 American Chemical Society.

In situ, DRIFTS IR spectra have suggested CO_3_^2−^ as an intermediate for CO formation and HCO_3_^−^ for CH_4_ (Figure 13a). Cui et al. worked on the roles of bridging ligands and metal ions grafted to AuNCs in the photocatalytic conversion of CO_2_ to CO [168]. The functionalization of L-cysteine with SG-protected Au nanoclusters (Au-GSH NCs) helped to bind metal cations such as Fe^2+^, Co^2+^, Ni^2+^, and Cu^2+^ and thus improved the selective CO production. Under visible light, along with CO_2_ and H_2_O, the Co^2+^ cation within the Au nanocluster (Auc-C-Co,) at an optimum concentration of 4 µmol, exhibited a maximum CO production of 3.45 µmol⋅gcat^−1^⋅h^−1^. Similarly, via 3-mercaptopropionic acid (MPA), Co^2+^ was grafted to the surface of the Au nanocluster (Au_c_-MPA-Co) and exhibited a steep rise in the photocatalytic activity compared to (Au_c_-C-Co) through the strong interlinkage between the S-metal cation (Figure 14).

**Figure 15:**
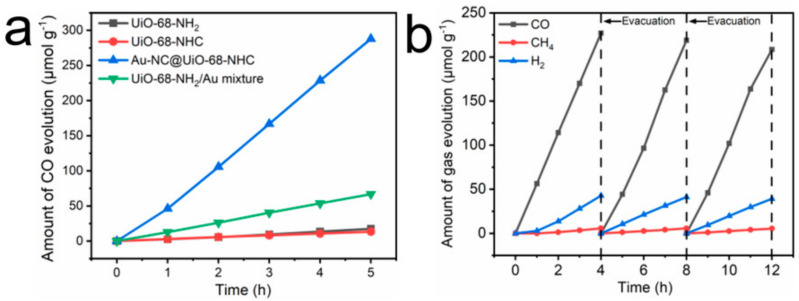
(**a**) CO evolution period of photocatalytic CO_2_ reduction using UiO-68-NHC, Au-NC@UiO-68-NHC, UiO-68-NH_2_, and Au/UiO-68-NH_2_ as photocatalysts upon AM 1.5G irradiation. (**b**) Time courses of photocatalytic CO_2_ reduction on Au-NC@ UiO-68-NHC under AM 1.5 G irradiation for 12 h, with evacuation every 4 h (dashed line). Reprinted with permission from [169] Copyright 2021 Wiley.

Zhang and co-workers [144] developed a quasi-ternary complex consisting of polymethacrylic-acid-stabilized Ag NCs (AgNCs-PMAA), carbon monoxide dehydrogenase (CODH), and TiO_2_ nanoparticles. The complex used as catalyst for visible light CO_2_ reduction, generated CO with a 20 s^−1^ turnover frequency at 25 °C. Jiang et al. [169] upgraded the chemical stability of AuNCs (Au-NCs) by combining them with a metal-organic framework (UiO-68) using N-Heterocyclic carbene-stabilizing ligands (NHC), denoted as AuNC@UiO-68-NHC. The photocatalytic activity of AuNC@UiO-68-NHC was enhanced due to the strong covalent bond formation between the AuNCs and UiO-68, which was facilitated by the Au-NHC bridges. This enabled the easy movement of excited electrons from the Fermi levels of the AuNCs to the conduction band of the UiO-68-NHC; thus, the recombination of the photogenerated electrons and holes was reduced (Figure 15) [169].

**Figure 16:**
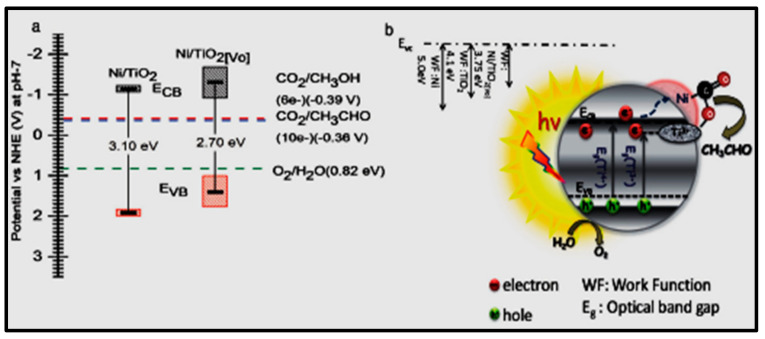
(**a**) Band edge positions. (**b**) Schematic illustration of photocatalytic CO_2_ reduction mechanism of Ni/TiO_2_[Vo]. Adapted from Ref [169].

The selective production of CO and the presence of CH_4_ and H_2_ as side products was observed. Billo and co-workers developed an effective photocatalyst with dual sites by making oxygen vacancies in Ni-NCs loaded on black TiO_2_ (Ni/TiO_2_[Vo]) [170]. Ni and oxygen defective sites act as dual sites, lessen the C-O bond strength, and support the separation of charge carriers (Figure 16). Under light irradiation from a halogen lamp, Ni/TiO_2_[Vo] produced 10 µmol g-cat^−1^ of acetaldehyde, 18 times higher than that of TiO_2_. Thus, the study referred to a different approach to enhancing photocatalytic CO_2_ reduction by introducing active dual sites into photocatalysts. Recently, El-Roz et al. prepared a silver-nanocluster-based catalyst for converting formic acid to CO_2_ and H_2_ under visible light irradiation. Here, the nanocluster was incorporated into a nano-sized zeolite crystal [169].

Table 1 summarizes the utilization of nanoclusters as a co-catalyst/catalysts for various photoreactions.

## 3. Conclusions and Future Perspective

MNCs are an under-explored class of nanomaterials with an unimaginable grade of potential. The review summarizes the recent progress in metal-nanocluster-based photocatalysts for various photocatalytic reactions, correlating it to their structure and properties, in order to boost the effectiveness of single metal and alloy hybrid systems and nanocomposite systems. However, research on MNCs has not surpassed the embryonic stage yet. In particular, preventing aggregation, effective capping agents or stabilizers, optical ajd electronic structures, metal-support bonding, mechanistic learning, in situ characterization techniques, structural model theories, and appreciable yields need more attention. Gold and AgNCs still dominate a major fraction of scientific publications in this domain; works on other transition metals could be more productive and cost-effective. The recyclability of MNCs in photocatalysis with an adequate stability after multiple cycles have to be extensively analyzed. At the same time, computer simulations could open up a world of possibilities for metal NCs to us, and recent additions to the software have proven their effectiveness. NCs can plausibly be the answer for low-carbon fuels, a milestone to sustainable development, and an affordable photocatalyst.

## Figures and Tables

**Table 1:** Summary of Literature Reports on the Use of nanocluster in Catalysis.

Sl NO	Photocatalyst	Co Catalyst	Application	Efficiency	Reference
1	TiO_2_-Au NCs@β-CD	AuNC coupled with per-6-thio-β-cyclodextrin (SH-β-CD)	Photodegradation of dyes	98% degradation in 10 min of exposure	[116]
2	Ag/TiO_2_/Nb	AgNC stabilized by Captopril	Photodegradation of dyes	100% degradation	[118]
3	AgNC/CeO_2_	AgNC stabilized by Polyethylene imine (PEI)	Photodegradation of dyes	80% degradation in 2 h	[119]
4	ZnO–Ag NCs.	AgNC	Photodegradation of dyes	100% degradation in 1 h	[120]
5	CuNC: [Cu_18_(CH_3_COO)(OH)]^−2^ and [Cu_34_O_2_(CH_3_COO)_3_N(C_4_H_9_)_3_Na]^−2.^	No cocatalyst	Photodegradation of dyes	100% degradation in 69 h	[121]
6	AuNC@MPTS (MPTS-3-Mercaptopropyl trimethoxysilane)	No cocatalyst	Photodegradation of dyes	100% degradation in 1 h	[49]
7	(Ag_12_TPyP)	No cocatalyst	Photodegradation of dyes	98% degradation	[123]
8	SiO_2_-Au GSH clusters-BPEI@TiO_2_	SiO_2_-Au GSH clusters-BEPI	Photodegradation of organic dyes	99.1% degradation in 0.5 h	[124]
9	Au_25_ NC-TiO_2_	Au_25_ NC	Oxidation of phenol derivatives and ferrocyanide and reduction of Ag^+^, Cu^2+^, and oxygen		[129]
10	Au_25_NC	No Catalyst	Oxidation of styrene and hydrogenation of α,β-unsaturated ketone	27 ± 1.0%	[130]
11	[Au_25_(PPh_3_)_10_(SR)_5_Cl_2_]-TiO_2_	AuNC: [Au_25_(PPh_3_)_10_(SR)_5_Cl_2_]	Oxidation of benzylamines to imines	73–99%	[126]
12	Ag/ZX-Bi_200	Ag NC: (Ag/ZX-Bi_200)	Photooxidation of methanol	49.60 mmol·g^−1^·cm^−2^ after 12 h of reaction	[132]
13	Aux-GSH NCs @TiO_2_	Aux-GSH NCs	Production of H_2_	0.3 mmol of hydrogen/h/g	[171]
14	Aux/NP-TNTA NP-TNTAs-TiO_2_ nanotube arrays	AuNC	Photodegradation of organic dyes, photocatalytic reduction of aromatic nitro compounds, and photoelectrochemical water splitting		[136]
15	(Au_25_(SG)_18_)-BaLa4Ti_4_O_15_	AuNC: (Au_25_(SG)_18_)	Photocatalytic water splitting	190 µmol/h	[146]
16	Au_25_/SrTiO_3_	AuNC	Hydrogen evolution reaction	41.2 µmol/h of H	[137]
17	Au_101_NCs-AlSrTiO_3_-rGO	Au_101_NCs	Photocatalytic production of H_2_, photocatalytic water splitting	385 ± 22 µmol h^−1^	[147]
18	GNRs-AgNCs GNRS-Gold nanorods	AgNCs	Hydrogen evolution reaction	10%	[138]
19	Pt/HTi_2_NbO_7_ Monolayer niobate (HTi_2_NbO_7_)	Pt NC	Higher H_2_ production	10 μmol h^−1^	[140]
20	Pt_46_NC-CdS Modified cadmium sulfide (CdS) nanorod	Pt_46_NC	Photocatalytic water splitting	1.5‰ h^−1^	[148]
21	Au_24_Pd NCs-BaLa_4_Ti_4_O_15_ & Au_24_Pt NCs- BaLa_4_Ti_4_O_15_	Au_24_Pd NCs and Au_24_Pt NCs	Photocatalytic H_2_ evolution	100–150 µmol h^−1^	[149]
22	PtAg_24_ NC-g-C_3_N_4_	PtAg_24_ NC	Photocatalytic H_2_ production	39.7 µmol h^−1^	[142]
23	Cu-BTN-TiO_2_	CuNCs: (Cu-BTN)	Photocatalytic CO_2_ reduction	150.9 μmol g^−1^ h^−1^	[167]
24	Metal cations-Fe^2+^, Co^2+^, Ni^2+^ and Cu^2+^	Au NCs: (Au_c_-C-Co) & (Au_c_-MPA-Co)	Photocatalytic CO_2_ reduction	3.45 µmol⋅g^−1^⋅h^−1^	[168]
25	CODH/AgNCs-PMAA/TiO_2_ CODH-carbon monoxide dehydrogenase	Ag NC coupled with PMAA	Photocatalytic CO_2_ reduction	turnover frequency of 20 s^−1^	[172]
26	Au-NCs@MOF		Photocatalytic CO_2_ reduction	57.6 μmol g^−1^ h^−1^	[169]
27	Ni-NCs-TiO_2_	Ni-NCs	Photocatalytic CO_2_ reduction	10 µmol g-cat^−1^	[170]
28	AgNC@ZX-V	No Cocatalyst	Reforming of formic acid to H_2_ and CO_2_	99% selectivity	[173]

## Data Availability

Not applicable.
